# MRI predictors of amyloid pathology: results from the EMIF-AD Multimodal Biomarker Discovery study

**DOI:** 10.1186/s13195-018-0428-1

**Published:** 2018-09-27

**Authors:** Mara ten Kate, Alberto Redolfi, Enrico Peira, Isabelle Bos, Stephanie J. Vos, Rik Vandenberghe, Silvy Gabel, Jolien Schaeverbeke, Philip Scheltens, Olivier Blin, Jill C. Richardson, Regis Bordet, Anders Wallin, Carl Eckerstrom, José Luis Molinuevo, Sebastiaan Engelborghs, Christine Van Broeckhoven, Pablo Martinez-Lage, Julius Popp, Magdalini Tsolaki, Frans R. J. Verhey, Alison L. Baird, Cristina Legido-Quigley, Lars Bertram, Valerija Dobricic, Henrik Zetterberg, Simon Lovestone, Johannes Streffer, Silvia Bianchetti, Gerald P. Novak, Jerome Revillard, Mark F. Gordon, Zhiyong Xie, Viktor Wottschel, Giovanni Frisoni, Pieter Jelle Visser, Frederik Barkhof

**Affiliations:** 1 Alzheimer Center & Department of Neurology, VU University Medical Center, PO Box 7057, 1007 MB Amsterdam, the Netherlands; 2 Laboratory of Epidemiology & Neuroimaging, IRCCS San Giovanni di Dio Fatebenefratelli, Brescia, Italy; 3 Alzheimer Centrum Limburg, Department of Psychiatry and Neuropsychology, Maastricht University, Maastricht, the Netherlands; 4 University Hospital Leuven, Leuven, Belgium; 5 Laboratory for Cognitive Neurology, Department of Neurosciences, KU Leuven, Leuven, Belgium; 6 AP-HM, CHU Timone, CIC CPCET, Service de Pharmacologie Clinique et Pharmacovigilance, Marseille, France; 7 Neurosciences Therapeutic Area Unit, GlaxoSmithKline R&D, Stevenage, UK; 8 U1171 Inserm, CHU Lille, Degenerative and Vascular Cognitive Disorders, University of Lille, Lille, France; 9 Sahlgrenska Academy, Institute of Neuroscience and Physiology, Section for Psychiatry and Neurochemistry, University of Gothenburg, Gothenburg, Sweden; 10 Barcelona βeta Brain Research Center, Pasqual Maragall Foundation, Barcelona, Spain; 11 Reference Center for Biological Markers of Dementia (BIODEM), Institute Born-Bunge, University of Antwerp, Antwerp, Belgium; 12 Department of Neurology and Memory Clinic, Hospital Network Antwerp (ZNA) Middelheim and Hoge Beuken, Antwerp, Belgium; 13 Neurodegenerative Brain Diseases, Center for Molecular Neurology, VIB, Antwerp, Belgium; 14 Laboratory of Neurogenetics, Institute Born-Bunge, University of Antwerp, Antwerp, Belgium; 15 Department of Neurology, Center for Research and Advanced Therapies, CITA-Alzheimer Foundation, San Sebastian, Spain; 16 Department of Psychiatry, University Hospital of Lausanne, Lausanne, Switzerland; 17 Geriatric Psychiatry, Department of Mental Health and Psychiatry, Geneva University Hospitals, Geneva, Switzerland; 18 Memory and Dementia Center, 3rd Department of Neurology, “G Papanicolau” General Hospital, Aristotle University of Thessaloniki, Thessaloniki, Greece; 19 University of Oxford, Oxford, UK; 20 King’s College London, London, UK; 21 Lübeck Interdisciplinary Platform for Genome Analytics, University of Lübeck, Lubeck, Germany; 22 School of Public Health, Imperial College London, London, UK; 23 Department of Psychology, University of Oslo, Oslo, Norway; 24 Department of Psychiatry and Neurochemistry, University of Gothenburg, Mölndal, Sweden; 25 Department of Molecular Neuroscience, UCL Institute of Neurology, Queen Square, London, UK; 26 UK Dementia Research Institute at UCL, London, UK; 27 Clinical Neurochemistry Laboratory, Sahlgrenska University Hospital, Mölndal, Sweden; 28 UCB Biopharma SPRL, Braine-l’Alleud, Belgium; 29 Janssen Pharmaceutical Research and Development, Titusville, NJ USA; 30 MAAT, Archamps, France; 31 Teva Pharmaceuticals, Inc., Malvern, PA USA; 32 Boehringer Ingelheim Pharmaceuticals, Inc., Ridgefield, CT USA; 33 Worldwide Research and Development, Pfizer Inc, Cambridge, MA USA; 34 Department of Radiology and Nuclear Medicine, VUMC, Amsterdam, the Netherlands; 35 University of Geneva, Geneva, Switzerland; 36 Institutes of Neurology and Healthcare Engineering, UCL, London, UK

**Keywords:** Alzheimer’s disease, Mild cognitive impairment, Biomarkers, Magnetic resonance imaging, Amyloid, Machine learning, Support vector machine, European Medical Information Framework for Alzheimer’s Disease

## Abstract

**Background:**

With the shift of research focus towards the pre-dementia stage of Alzheimer’s disease (AD), there is an urgent need for reliable, non-invasive biomarkers to predict amyloid pathology. The aim of this study was to assess whether easily obtainable measures from structural MRI, combined with demographic data, cognitive data and apolipoprotein E (*APOE*) ε4 genotype, can be used to predict amyloid pathology using machine-learning classification.

**Methods:**

We examined 810 subjects with structural MRI data and amyloid markers from the European Medical Information Framework for Alzheimer’s Disease Multimodal Biomarker Discovery study, including subjects with normal cognition (CN, *n* = 337, age 66.5 ± 7.2, 50% female, 27% amyloid positive), mild cognitive impairment (MCI, *n* = 375, age 69.1 ± 7.5, 53% female, 63% amyloid positive) and AD dementia (*n* = 98, age 67.0 ± 7.7, 48% female, 97% amyloid positive). Structural MRI scans were visually assessed and Freesurfer was used to obtain subcortical volumes, cortical thickness and surface area measures. We first assessed univariate associations between MRI measures and amyloid pathology using mixed models. Next, we developed and tested an automated classifier using demographic, cognitive, MRI and *APOE* ε4 information to predict amyloid pathology. A support vector machine (SVM) with nested 10-fold cross-validation was applied to identify a set of markers best discriminating between amyloid positive and amyloid negative subjects.

**Results:**

In univariate associations, amyloid pathology was associated with lower subcortical volumes and thinner cortex in AD-signature regions in CN and MCI. The multi-variable SVM classifier provided an area under the curve (AUC) of 0.81 ± 0.07 in MCI and an AUC of 0.74 ± 0.08 in CN. In CN, selected features for the classifier included *APOE* ε4, age, memory scores and several MRI measures such as hippocampus, amygdala and accumbens volumes and cortical thickness in temporal and parahippocampal regions. In MCI, the classifier including demographic and *APOE* ε4 information did not improve after additionally adding imaging measures.

**Conclusions:**

Amyloid pathology is associated with changes in structural MRI measures in CN and MCI. An automated classifier based on clinical, imaging and *APOE* ε4 data can identify the presence of amyloid pathology with a moderate level of accuracy. These results could be used in clinical trials to pre-screen subjects for anti-amyloid therapies.

**Electronic supplementary material:**

The online version of this article (10.1186/s13195-018-0428-1) contains supplementary material, which is available to authorized users.

## Background

Alzheimer’s disease (AD) is characterized pathologically by beta-amyloid (Aβ) plaques and neurofibrillary tangles of misfolded tau protein [1]. As amyloid pathology may arise up to two decades before the onset of dementia, research focus has shifted towards the pre-dementia stage, which provides an opportunity for secondary prevention [2–4]. The design of clinical trials targeting the amyloid pathway in this early stage would be facilitated by the ability to recruit subjects with amyloid pathology. Amyloid pathology can be assessed in cerebrospinal fluid (CSF), obtainable by lumbar puncture, or on positron emission tomography (PET) scans. However, obtaining CSF is relatively invasive and PET scans are costly, invasive by exposing subjects to radiation and are not universally available. As the estimated prevalence of amyloid pathology between the ages of 60 and 80 ranges from 10 to 33% for cognitively normal (CN) subjects and from 37 to 60% for subjects with mild cognitive impairment (MCI) [5], assessing amyloid pathology with CSF or PET for screening purposes is likely inefficient. Finding minimally invasive biomarkers predicting amyloid pathology could reduce the number of invasive, costly and time-consuming measures in clinical trials.

Brain atrophy markers derived from structural magnetic resonance imaging (MRI) could serve as a potential biomarker for amyloid pathology [6–12]. In this study, we evaluate the use of easily obtainable MRI measures for the prediction of amyloid pathology. We included both visual rating scores, which can be easily performed in clinical settings, and quantitative measures of subcortical volumes, cortical thickness and surface area, which can be derived from freely available software and may be more sensitive than visual ratings. We first assessed univariate associations between MRI measures and amyloid pathology. Next, we used support vector machine (SVM) analysis to develop a multi-variable classifier for predicting brain amyloid pathology at a single subject level. Besides imaging measures, we also included other non-invasive measures relevant to AD in the classifier, including demographic information, cognitive testing and apolipoprotein E (*APOE*) ε4 genotype.

## Methods

### Participants

We included participants from the European Medical Information Framework for Alzheimer’s Disease Multimodal Biomarker Discovery (EMIF-AD MBD) study. The aim of this study was to discover novel diagnostic and prognostic markers for pre-dementia AD, by making use of existing data and samples [13]. The EMIF-AD MBD study pooled data of 494 CN, 526 MCI and 201 AD-dementia participants from three multicentre and eight single-centre studies. Inclusion criteria were: presence of normal cognition, MCI or a clinical diagnosis of AD-type dementia; availability of data on amyloid pathology, measured in CSF or on PET; age above 50 years; availability of MRI scans, plasma, DNA or CSF samples (at least two of the modalities); and absence of major neurological, psychiatric or somatic disorders that could cause cognitive impairment.

**Fig. 1:**
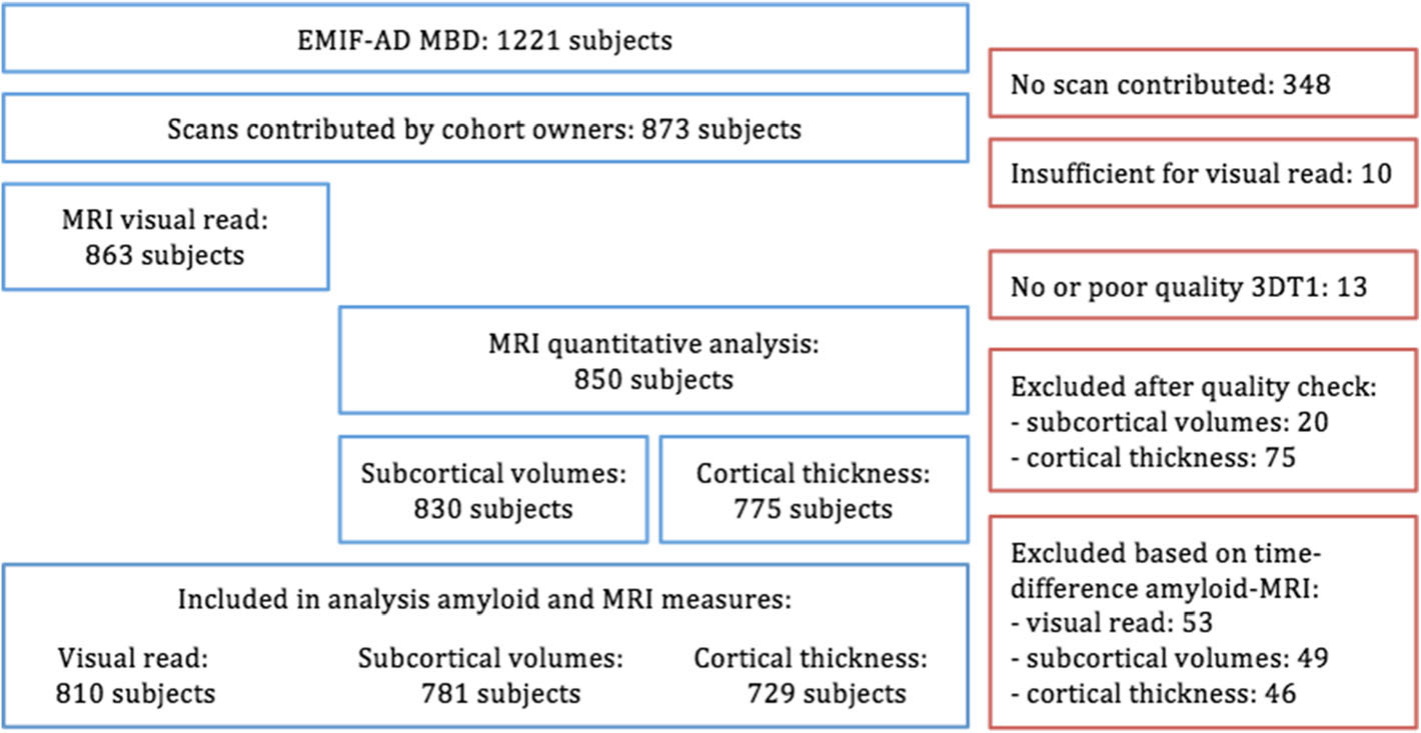
Number of included subjects. *EMIF-AD MBD* European Medical Information Framework for Alzheimer’s Disease Multimodal Biomarker Discovery, *MRI* magnetic resonance imaging

From the 1221 subjects included in the EMIF-AD MBD study, MRI scans of 873 subjects were contributed by the different studies (Fig. 1). Based on visual assessment, 863 MRI scans were of sufficient quality for visual rating, consisting of 365 CN, 398 MCI and 100 AD-dementia participants. Data were obtained from the following cohorts: DESCRIPA [14], EDAR [15], PharmaCog [16] and single-centre studies at VU University Medical Centre [17], San Sebastian GAP [18], University of Antwerp [19], Leuven [20], University of Lausanne [21], University of Gothenburg [22] and Barcelona IDIBAPS [23]. Each study was approved by the local medical ethics committee. Subjects had provided written informed consent at the time of inclusion in the MBD study for sharing of data, fluid samples and scans.

### Clinical and cognitive data

From all parent cohorts, clinical information and neuropsychological tests were collected centrally, harmonized, pooled and stored in an online data platform as previously described [13]. In short, all parent cohorts administered the Mini-Mental State Examination (MMSE), and performed neuropsychological testing covering various cognitive domains, although the tests used varied across the different cohorts. For the cognitive domains memory, language, attention, executive functioning and visuo-construction, one priority test was selected from each cohort (Additional file 1: Table S1) and *z*-scores were computed based on local normative data when available, or published normative data from healthy controls otherwise.

### APOE genotyping

For the entire EMIF-AD MBD cohort, *APOE* genotyping data from the local genetic analyses were available for 1121 (91%) individuals. Central genetic analyses were performed at Lübeck University, Germany for 805 DNA and 148 whole blood samples. From the blood samples, DNA was extracted using the QIAamp® DNA Blood Mini Kit (QIAGEN GmbH, Hilden, Germany) resulting in 953 DNA samples, of which 926 passed quality control. Genome-wide SNP genotyping was performed using the Infinium Global Screening Array (GSA) with Shared Custom Content (Illumina Inc.). *APOE* genotypes were determined either directly (rs7212) or by imputation (rs429358). For 80 samples for which no local *APOE* genotype was available, and for 45 mismatches between local and GSA-derived genotypes, the *APOE* genotype was determined using TaqMan assays (ThermoFisher Scientific, Foster City, CA, USA) on a QuantStudio-12 K-Flex system. TaqMan re-genotyping confirmed 23 GSA genotypes and 21 local genotypes. For one failed sample we retained the local genotype. We classified individuals as *APOE* ε4 carriers or non-carriers according to their genotype status at rs429358 (C-allele = ε4).

### Amyloid classification

In the current selection (*n* = 863), amyloid status was defined by central analysis of CSF when available (*n* = 510), otherwise by local amyloid PET (*n* = 174) or local CSF (*n* = 179) measures. Central CSF analysis was performed at Gothenburg University, Sweden and included Aβ_1–40_ and Aβ_1–42_ measured using the V-PLEX Plus Aβ Peptide Panel 1 (6E10) Kit (Meso Scale Discovery, Rockville, MD, USA), as described by the manufacturer. The central cut-off value for Aβ positivity was an Aβ_42/40_ ratio < 0.061. Amyloid PET was performed in one cohort using [^18^F]flutemetamol according to local standardized procedures, with a standardized uptake value ratio (SUVR) cut-off value > 1.38 used for abnormality [24]. In short, SUVR images were computed from spatially normalized summed images with cerebellar grey matter as the reference region. The cut-off value was derived from an independent dataset [25] and based on the statistical difference between AD dementia patients and cognitively normal subjects [24]. Local CSF amyloid was determined according to local protocols with local cut-off values. The number of amyloid positive subjects per diagnosis per cohort is presented in Additional file 1: Table S2.

### MRI acquisition

At each site, imaging was acquired according to local protocols. From each parent cohort, we centrally collected the T1-weighted images, and if available also fluid-attenuated inversion recovery (FLAIR) and susceptibility weighted images (SWI) or T2*, at the VU University Medical Center, where a visual quality check was performed. The acquired sequences and acquisition parameters for the T1-weighted scans for each cohort are presented in Additional file 1: Table S3. Usually, MRI was assessed at baseline together with baseline cognitive and amyloid measures. For 104 subjects there was more than a 1-year difference between MRI acquisition and amyloid assessment. In cases where amyloid was abnormal and acquired before MRI, this subject was included in the analysis (*n* = 42). In cases where amyloid was normal and acquired after MRI, this subject was included in the analysis (*n* = 9). All other cases were excluded (*n* = 53). For 99 subjects there was more than a 1-year difference between baseline cognitive assessment and MRI. For these cases, we did not use the cognitive data in the multi-variable analysis. Demographic differences between subjects who were included and excluded for differences in time between MRI and amyloid or cognitive assessment are presented in Additional file 1: Tables S4 and S5.

### MRI visual rating

MRI scans with sufficient quality (*n* = 863) were visually rated by a single experienced rater, blinded to demographic information during rating. Medial temporal lobe atrophy (MTA) was assessed on coronal reconstructions of the T1-weighted images using a 5-point scale ranging from no atrophy (0) to end-stage atrophy (4) [26]. The MTA results from the left and right hemisphere were averaged. Global cortical atrophy (GCA) was assessed on transversal FLAIR or T1 images using a 4-point scale [27]. Posterior atrophy was assessed using a 4-point scale [28] and averaged over hemispheres. White matter hyperintensities were visually assessed on FLAIR images (*n* = 812) using the 4-point Fazekas scale (none, punctate, early confluent, confluent) [29]. Microbleeds were assessed on SWI and/or T2* images (*n* = 445) and defined as rounded hypointense homogeneous foci of up to 10 mm in diameter in the brain parenchyma. Microbleeds were dichotomized as present (≥ 1 microbleeds) or absent (0 microbleeds).

### MRI quantitative analysis

Good quality 3D T1 images (*n* = 850) were uploaded on the N4U platform (https://neugrid4you.eu/) for automated quantitative processing. Subcortical volumes, cortical thickness and surface area measures were estimated from 3D T1 MRI using Freesurfer (version 5.3.0, https://surfer.nmr.mgh.harvard.edu) as previously described [30]. All segmentations were visually inspected. We excluded data from 20 subjects for subcortical volumes (five due to complete failure of the algorithm and 15 due to segmentation errors) and from 75 subjects for cortical thickness and surface area (five due to complete failure of the algorithm, 66 due to segmentations errors of the cortical ribbon and four for other failures). Subcortical volumes were normalized by total intracranial volume (TIV). Cortical thickness and surface area were available for 68 regions according to the Desikan–Killiany atlas implemented in Freesurfer. Additionally, we computed two AD-signature meta-ROI measures that have previously been presented in the literature: one by Dickerson et al. [10] consisting of the average cortical thickness in angular, precuneus, supramarginal, superior frontal, superior parietal, temporal pole, inferior temporal, medial temporal and inferior frontal cortex; and one by Jack et al. [31] consisting of the surface-area weighted average mean cortical thickness in entorhinal, inferior temporal, middle temporal and fusiform regions.

### Statistical methods

#### Univariate analysis

Univariate statistical analyses were performed in R (version 3.3.1). Comparisons of clinical characteristics between amyloid positive and negative subjects within each diagnostic group were performed using independent *t* tests or Mann–Whitney *U* tests for continuous variables and chi-square tests for categorical variables. Baseline comparisons in quantitative MRI measures between groups were performed with linear mixed models (continuous outcome measures) (lme4 package, version 1.1–12; lmerTest package 2.0–36), mixed effects ordered logistic regressions (ordinal outcome measures) (ordinal package, version 2015.6–28) and mixed effects logistic regressions (dichotomous outcome measures) (lme4 package). In each model, we entered amyloid status (negative, positive) and diagnosis (CN, MCI and AD) and their interaction as fixed effects. Age (centred on mean), gender and *APOE* ε4 status were added as covariates. Cohort was added as a random intercept. The analyses were corrected within diagnostic group (in total 22 tests: five visual ratings, 14 subcortical volumes, three cortical thickness summary measures) for multiple hypothesis testing with the p.adjust() function using the false discovery rate, and indicated as *p*_FDR_.

#### Multi-variable analysis

To find the best multi-variable predictor of amyloid pathology, we used a supervised machine-learning approach based on SVM analysis. In SVM, two classes are separated by finding a hyperplane that maximizes the margin of separation between data points of each class in a high-dimensional feature space. SVMs are used extensively in neuroimaging as they have been shown to predict outcomes with high accuracy and possess the ability to model diverse and high-dimensional data [32]. We built a classifier to separate amyloid positive from amyloid negative subjects separately in the CN and MCI subgroups and, for the sake of completeness, also in the whole sample (including CN, MCI and AD-dementia patients). To address the imbalance between the number of amyloid positive and amyloid negative subjects in each diagnostic group, we adopted the re-weighting strategy [33]. That means we adjusted weights of each SVM feature inversely proportional to amyloid positive versus negative frequencies.

##### Machine-learning approach

We used the python Scikit-learn library (version 0.19.1) to perform SVM classification [34]. To prevent overfitting (i.e. the classifier works perfectly on the training data, but is poorly generalizable to new data), we performed feature relevance evaluation and dimensionality reduction using a tree-based feature selection approach with a nested 10-fold cross-validation design [35, 36]. This was performed separately within each subgroup (CN, MCI and whole sample).

The nested cross-validation consists of an inner loop for model building and parameter estimation, and an outer loop for model testing. Consequently, the dataset was divided into two parts: a training plus validation subset and a test subset. In the inner loop, SVM models were trained with varying SVM hyper-parameters (i.e. cost parameters *C* and kernel function) based on a grid search, and a feature selection was performed using classification trees. The validation set was used to determine the SVM hyper-parameters over the grid of possible values. The performance of the resulting model, with optimized SVM hyper-parameters and features, was subsequently evaluated on the test set in the outer loop. For this outer loop, we used a 10-fold cross-validation scheme so that the data were divided into 10 equally sized parts. Nine of these were used as the training/validation set and one as the test set, and the 10 parts were permuted in each iteration of the outer loop so that each one was used for testing once. Finally, the SVM results were averaged over the 10 folds to estimate the predictive power of the proposed model on the whole dataset.

##### Feature selection

As the input for the classifier, we used demographic information, neuropsychological information, *APOE* ε4 genotype and MRI measures (visual ratings, subcortical volumes, regional cortical thickness and regional surface area measures). To combine information measured on different scales, continuous demographic and MRI measures were normalized to *z*-scores. In the adopted tree-based feature selection strategy, the Gini index was used to measure the relevance of each feature [37]. Features with a Gini index above the mean were kept, others were discarded. The complete list of features considered and selected, in the whole dataset and for CN and MCI separately, is reported in Additional file 1: Table S8.

##### Performance evaluation

To assess the performance of the classifier, we computed the averaged receiver operating characteristic (ROC) area under the curve (AUC), specificity, sensitivity and accuracy for the testing datasets. We initially maximized the Youden index, and then also explored the results when setting the sensitivity at 80%, 85%, 90%, 95% and 100%. To assess the added value of combining different sources of information, we also built classifiers including only demographic information and a single other biomarker type (neuropsychological tests, *APOE* ε4 genotype, MRI measures). Differences in AUC ROCs between classifiers were assessed with DeLong’s test.

## Results

### Demographic and cognitive comparisons

We included 810 subjects divided over three diagnostic groups: CN (*n* = 337), MCI (*n* = 375) and AD dementia (*n* = 98). Within the CN group, 92 (27%) subjects were amyloid positive, in the MCI group 235 (63%) and in the AD-dementia group 95 (97%). Demographic and clinical data according to diagnosis and amyloid status are presented in Table 1. The amyloid positive MCI subjects were older and had lower cognitive scores compared to the amyloid negative MCI subjects. In CN, there were no differences in age or cognition between amyloid positive and amyloid negative subjects. Amyloid positive subjects were more often *APOE* ε4 carriers in both the MCI and CN groups.

**Table 1:** Baseline characteristics by diagnosis and amyloid status

Characteristic	Cognitively normal		Mild cognitive impairment		Alzheimer-type dementia	
	Amyloid negative	Amyloid positive	Amyloid negative	Amyloid positive	Amyloid negative	Amyloid positive
*N*, % within diagnosis	245 (73)	92 (27)	140 (37)	235 (63)	3 (3)	95 (97)
Age (years)	66.1 ± 7.2	67.5 ± 7.2	67.3 ± 8.0	70.2 ± 7.0***	63.1 ± 8.0	67.1 ± 7.7
Male gender	120 (49)	47 (51)	73 (52)	105 (45)	3 (100)	48 (51)
Education (years)	13.2 ± 3.5	12.8 ± 3.8	10.8 ± 4.0	11.1 ± 3.7	10.3 ± 5.1	11.1 ± 3.3
MMSE	28.9 ± 1.2	28.8 ± 1.2	27.1 ± 2.2	26.0 ± 2.6***	27.7 ± 1.2	22.4 ± 4.0**
Memory immediate	0.10 ± 1.00	0.08 ± 1.10	− 0.64 ± 1.32	−1.22 ± 1.44***	−0.45 ± 0.91	−2.25 ± 1.06
Memory delayed	0.25 ± 1.01	0.30 ± 1.09	−0.90 ± 1.29	− 1.37 ± 1.41**	−0.96 ± 1.33	− 2.28 ± 1.04
Language	−0.21 ± 1.01	0.01 ± 1.04	−0.65 ± 1.30	−0.88 ± 1.27	−0.76 ± 0.38	−1.95 ± 1.02*
Attention	0.32 ± 1.03	0.26 ± 0.89	−0.74 ± 1.79	−0.81 ± 1.63	0.54 ± 0.52	− 2.03 ± 1.94*
Executive functioning	0.35 ± 1.09	0.12 ± 1.15	− 0.76 ± 1.89	−1.11 ± 1.98*	0.46 ± 0.32	−2.49 ± 2.46*
Visuo-construction	− 0.23 ± 1.36	− 0.19 ± 1.20	0.18 ± 1.46	−0.30 ± 1.66*	−0.59 ± 2.09	−1.30 ± 2.00
*APOE* ε4 genotype	89 (36)	53 (58)***	27 (19)	160 (66)***	2 (67)	66 (69)
Available markers						
Visual	245 (100)	92 (100)	140 (100)	235 (100)	3 (100)	95 (100)
Subcortical volumes	240 (98)	90 (98)	130 (93)	230 (98)*	2 (67)	89 (94)
Cortical thickness	232 (95)	88 (96)	119 (85)	200 (85)	2 (67)	88 (93)

Data presented as mean ± standard deviation or count (%). Demographic characteristics based on maximum available data (visual rating)

*APOE* apolipoprotein E, *MMSE* Mini-Mental State Examination

*p < 0.05, **p < 0.01, ***p < 0.001, difference between amyloid positive and negative within diagnostic group

### Univariate association between MRI measures and amyloid pathology

Within the MCI group, subjects with amyloid pathology had higher visual rating scores of medial temporal lobe atrophy, global cortical atrophy and parietal atrophy compared to amyloid negative subjects (Table 2). There were no differences in Fazekas score or presence of microbleeds. Amyloid positive MCI subjects had statistically significantly lower bilateral hippocampus, amygdala, thalamus, left caudate and right putamen volumes, and a trend towards lower right caudate (*p*_uncorrected_ = 0.08) and bilateral accumbens (both *p*_uncorrected_ = 0.07) volumes compared to amyloid negative MCI subjects (Table 3). Amyloid positive MCI subjects also had lower whole brain average cortical thickness, as well as in the two AD-signature meta-ROIs, compared to amyloid negative MCI subjects.

**Table 2:** Visual rating scores according to diagnosis and amyloid status

Score	Cognitively normal		Mild cognitive impairment		Alzheimer-type dementia	
	Amyloid negative	Amyloid positive	Amyloid negative	Amyloid positive	Amyloid negative	Amyloid positive
MTA	0 (0–1)	0 (0–1)	0.5 (0–1)	1 (0.5–1.5)^††^	1 (0.5–2)	1 (1–2)
GCA	0 (0–1)	0 (0–1)	0 (0–1)	1 (0–1)^††^	1 (0–2)	1 (1–1)
Parietal	1 (0–1)	0.5 (0–1)	1 (0–1)	1 (0–1.63)^†^	2 (0–2)	1 (1–2)
Fazekas	1 (0–1)	1 (0–1)	1 (0–1)	1 (1–2)	1 (1–1)	1 (0–2)
Microbleeds present	6 (21%)	4 (20%)	29 (25%)	56 (29%)	1 (50%)	17 (22%)

Data presented as median (interquartile range) or count (%)

*APOE* apolipoprotein E, *FDR* false discovery rate, *GCA* global cortical atrophy, *MTA* medial temporal lobe atrophy

^†^*p*_FDR_ < 0.05, ^††^*p*_FDR_ < 0.01, difference between amyloid positive and negative within diagnostic group. Analyses corrected for age, gender, *APOE* ε4 genotype and cohort

**Table 3:** Quantitative MRI measures according to diagnosis and amyloid status

MRI measure	Cognitively normal		Mild cognitive impairment		Alzheimer-type dementia		*F* value		
	Amyloid negative	Amyloid positive	Amyloid negative	Amyloid positive	Amyloid negative	Amyloid positive	Diagnosis	Amyloid	Diagnosis × amyloid
Hippocampus left	3837 (39)	3752 (58)	3638 (46)	3353 (47)^†††^	3051 (340)	3124 (61)	32.0***	0.7	3.5*
Hippocampus right	3960 (53)	3830 (66)^#^	3760 (57)	3389 (57)^†††^	3905 (337)	3172 (70)^#^	19.7***	12.3***	5.4**
Amygdala left	1501 (36)	1439 (40)^#^	1405 (37)	1294 (37)^†††^	1604 (171)	1188 (42)^#^	9.2***	11.0**	2.5
Amygdala right	1567 (52)	1535 (55)	1522 (53)	1398 (53)^†††^	1612 (183)	1290 (57)	4.6**	6.7**	3.2*
Thalamus left	6834 (101)	6614 (119)^#^	6951 (107)	6689 (108)^†^	6187 (564)	6787 (126)	1.1	0.04	1.1
Thalamus right	6388 (104)	6320 (113)	6419 (106)	6173 (107)^†††^	5600 (420)	6185 (117)	2.4	0.4	3.2*
Caudate left	3419 (67)	3336 (80)	3571 (71)	3407 (71)^†^	4151 (393)	3387 (85)	2.9	6.1*	1.7
Caudate right	3491 (88)	3396 (98)	3584 (91)	3463 (91)	4575 (407)	3413 (103)^#^	3.7*	10.7**	3.4*
Putamen left	4831 (105)	4779 (117)	4689 (108)	4609 (109)	4692 (478)	4509 (122)	2.1	0.4	0.1
Putamen right	4627 (121)	4607 (130)	4659 (123)	4461 (124)^†^	4524 (444)	4302 (134)	0.6	0.9	1.5
Pallidum left	1366 (32)	1412 (37)	1370 (33)	1385 (33)	1488 (168)	1390 (39)	0.3	0.05	0.6
Pallidum right	1388 (26)	1382 (31)	1384 (28)	1379 (28)	1361 (148)	1392 (33)	0.02	0.01	0.03
Accumbens left	465 (23)	432 (24)^#^	434 (23)	411 (23)	526 (79)	375 (25)	2.1	6.7**	1.5
Accumbens right	497 (22)	467 (23)^#^	466 (22)	443 (22)	425 (78)	419 (24)	2.3	4.0*	0.6
Average CT	2.29 (0.02)	2.28 (0.02)	2.27 (0.02)	2.22 (0.02)^†††^	2.22 (0.07)	2.19 (0.02)	5.3**	1.6	1.5
CT Dickerson	2.54 (0.02)	2.52 (0.02)	2.50 (0.02)	2.45 (0.02)^††^	2.48 (0.09)	2.38 (0.02)	8.3***	3.5	1.6
CT Jack	2.68 (0.03)	2.63 (0.03)^#^	2.63 (0.03)	2.56 (0.03)^†††^	2.67 (0.10)	2.47 (0.03)^#^	6.7**	9.3**	1.7

Data presented as estimate (standard error). Estimates derived from linear mixed models including diagnosis × amyloid, age, gender and *APOE* ε4 genotype as covariates and cohort as random effect

*APOE* apolipoprotein E, *CT* cortical thickness, *FDR* false discovery rate, *MRI* magnetic resonance imaging

^†^*p*_FDR_ < 0.05, ^††^*p*_FDR_ < 0.01, ^†††^*p*_FDR_ < 0.001, ^#^*p*_uncorrected_ < 0.05, compared to amyloid negative within diagnostic group

*p < 0.05, **p < 0.01, ***p < 0.001 for *F* statistic of main effect

In the CN group, amyloid positive subjects had statistically significantly lower right hippocampus, left amygdala, left thalamus and bilateral accumbens volumes compared to amyloid negative subjects. The effect of amyloid pathology on hippocampal volume was stronger in MCI subjects compared to CN subjects (significant interaction diagnosis × amyloid status). Amyloid positive CN subjects had lower values in the Jack AD-signature meta-ROI (*p*_uncorrected_ = 0.02), but not in the Dickerson AD-signature meta-ROI (*p*_uncorrected_ = 0.3) or whole brain average cortical thickness (*p*_uncorrected_ = 0.3) compared to amyloid negative CN subjects. There were no differences in visual rating scores between amyloid positive and amyloid negative CN subjects. All individual cortical thickness and surface area regions are presented in Additional file 1: Tables S6 and S7.

Compared to amyloid positive CN subjects, amyloid positive MCI subjects had lower bilateral hippocampal and amygdala volumes (all *p* < 0.001) and lower whole brain average cortical thickness (*p* = 0.001), as well as in the two AD-signature meta-ROIs (both *p* < 0.001).

### Multi-variable classifier results

**Fig. 2:**
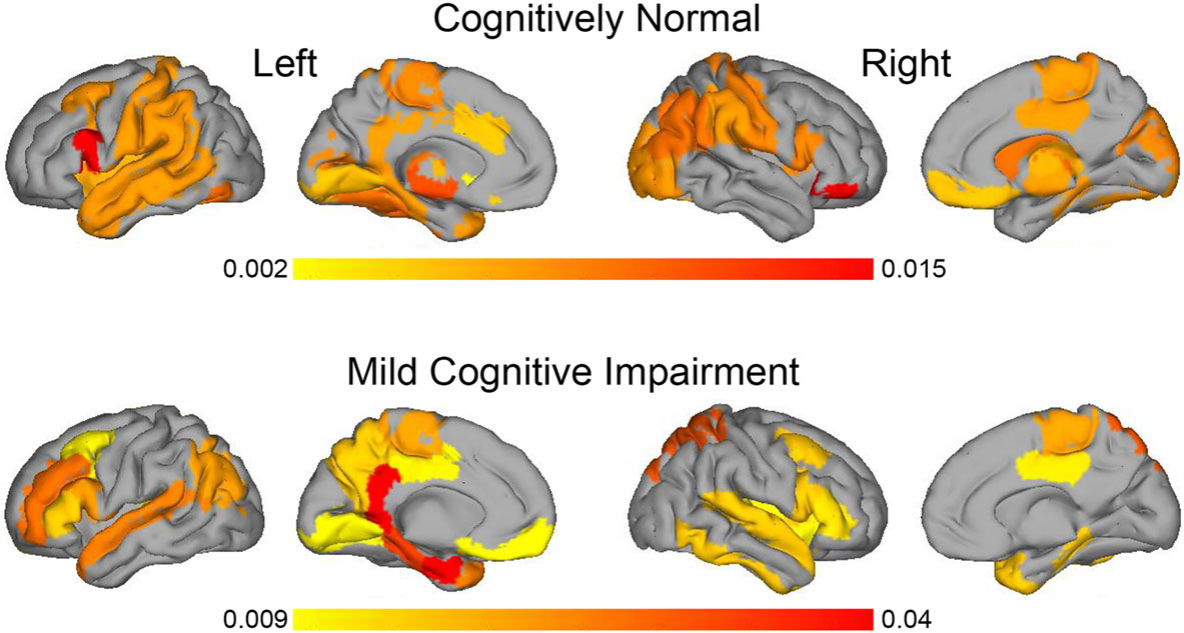
Freesurfer regions selected as features for the classifier in cognitively normal (top row) and mild cognitive impairment (bottom row). Colour bars represent averaged feature weight

The features selected by the classifier in CN subjects, MCI subjects and the whole sample are presented in Additional file 1: Table S8. Across diagnoses, *APOE* ε4 genotype was the most important feature. Other relevant features selected across samples were age, the neuropsychological memory scores and various MRI measures such as hippocampus and amygdala volumes, as well as cortical thickness in temporal and parahippocampal regions (Fig. 2).

**Fig. 3:**
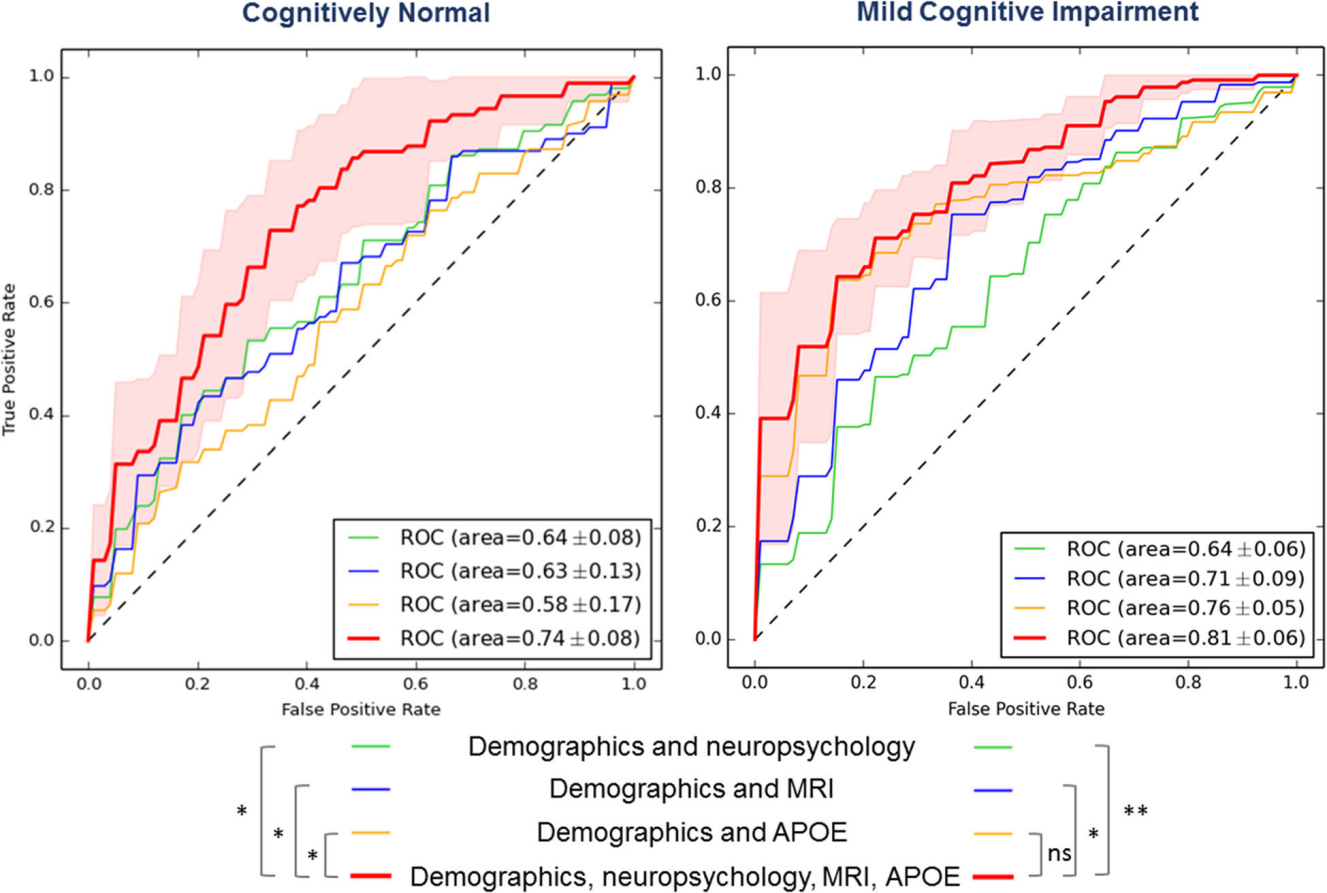
Classifier results. Receiver operating characteristic (ROC) curves of support vector machine classifier to predict amyloid pathology in cognitively normal (left panel) and mild cognitive impairment (right panel) subjects. Red: results from the combined classifier, including demographic information, neuropsychological tests, MRI measures and *APOE* ε4 genotype. Specific features selected presented in Additional file 1: Table S8. Classifier results from demographic information combined with only neuropsychology (green), or MRI measures (blue) or *APOE* ε4 genotype (yellow). ROC significant differences assessed with DeLong’s test. *p < 0.05, **p < 0.001, ns not significant. *APOE*, apolipoprotein E, MRI magnetic resonance imaging

Combining the informative selected features in the SVM resulted in AUC = 0.81 ± 0.06 in MCI subjects, AUC = 0.74 ± 0.08 in CN subjects and AUC = 0.85 ± 0.05 in the whole sample to classify amyloid positive versus amyloid negative subjects (Fig. 3; Additional file 2: Figure S1). In MCI, the combined classifier including information from all modalities performed statistically significantly better than the classifiers based on demographic information combined with neuropsychology or imaging measures alone. The classifier including demographic variables and *APOE* ε4 genotype did not improve after additionally adding imaging and cognitive variables in MCI. In CN, the combined classifier including information from all modalities (demographics, cognitive, genetics and imaging) performed statistically significantly better than the classifiers including variables from only a subset of these modalities (Fig. 3; Additional file 2: Figure S2). The results from the SVM including only imaging variables are displayed in Additional file 2: Figure S3.

Table 4 presents the accuracy, sensitivity and specificity of the combined SVM in CN subjects, MCI subjects and the whole sample maximizing the Youden index, and at different levels of sensitivity. When optimizing both specificity and sensitivity, the positive predictive value for amyloid pathology was 0.84 in MCI (a 42% increase compared to the a priori probability (i.e. prevalence) of 0.59) and the negative predictive value was 0.62. In CN, the SVM obtained a positive predictive value of 0.41 (a 64% increase compared to the prevalence of 0.25) and a negative predictive value of 0.84.

**Table 4:** Sensitivity, specificity, accuracy, PPV and NPV of the SVM classifier

Group	Sensitivity	Specificity	Accuracy	PPV	NPV	Threshold SVM
Optimized sensitivity and specificity						
Cognitively normal	0.61	0.71	0.68	0.41	0.84	0.70
Mild cognitive impairment	0.71	0.77	0.74	0.84	0.62	0.33
Whole sample	0.75	0.79	0.77	0.80	0.74	0.48
80% sensitivity						
Cognitively normal	0.80	0.55	0.62	0.41	0.88	0.77
Mild cognitive impairment	0.80	0.64	0.74	0.79	0.66	0.47
Whole sample	0.80	0.69	0.75	0.74	0.76	0.56
85% sensitivity						
Cognitively normal	0.85	0.46	0.57	0.38	0.89	0.80
Mild cognitive impairment	0.85	0.54	0.73	0.75	0.68	0.53
Whole sample	0.85	0.59	0.73	0.69	0.78	0.64
90% sensitivity						
Cognitively normal	0.90	0.36	0.51	0.35	0.91	0.84
Mild cognitive impairment	0.90	0.46	0.73	0.73	0.74	0.60
Whole sample	0.90	0.51	0.71	0.67	0.83	0.70
95% sensitivity						
Cognitively normal	0.95	0.24	0.44	0.32	0.92	0.87
Mild cognitive impairment	0.95	0.37	0.73	0.71	0.83	0.70
Whole sample	0.95	0.40	0.69	0.63	0.88	0.79
100% sensitivity						
Cognitively normal	1.00	0.11	0.36	0.30	1.00	0.91
Mild cognitive impairment	1.00	0.08	0.66	0.64	1.00	0.87
Whole sample	1.00	0.04	0.54	0.53	1.00	0.95

Results from combined classifier, including demographic information, neuropsychological tests, MRI measures and *APOE* ε4 genotype. Specific features selected presented in Additional file 1: Table S8. Values averaged across 10-fold cross-validation. Youden’s *J* statistic employed

*APOE* apolipoprotein E, *MRI* magnetic resonance imaging, *NPV* negative predictive value, *PPV* positive predictive value, *SVM* support vector machine

## Discussion

In this study, we found that amyloid pathology is associated with brain atrophy in CN and MCI subjects. Using machine-learning techniques, we built a classifier based on a combination of demographic, cognitive, *APOE* ε4 genotype and MRI data that could predict amyloid status at single subject level with a moderate level of accuracy. The performance of the classifier was higher in MCI subjects than in CN subjects. These results are of interest for clinical trial designers who wish to recruit amyloid positive subjects for inclusion.

Our results on the association between amyloid pathology and MRI measures in MCI are in line with previous studies that also found more cortical and subcortical atrophy in amyloid positive compared to amyloid negative MCI subjects [6, 7]. In CN, amyloid pathology has previously been associated with cortical atrophy [9–11], and lower hippocampal volume in some studies [8, 9], but not in all [11, 38]. To capture cortical changes associated with AD, two different AD-signature meta-ROIs have been proposed in the literature [10, 31]. In MCI, both AD-signature measures were related to amyloid pathology. In CN, only the AD-signature meta-ROI by Jack et al. [31] was associated with amyloid pathology in our study, suggesting that this one is more sensitive in the early disease stage. We also found an effect of amyloid pathology on nucleus accumbens volume, which was most pronounced in CN subjects. Although nucleus accumbens volumes are not often measured in AD-related studies, it has been hypothesized that this structure could show secondary neurodegeneration in AD in response to reduced input from connections to medial-temporal lobe structures [39]. It should be noted, however, that the nucleus accumbens is a small structure, which is difficult to segment automatically. These results require further validation in future studies.

The optimal features selected in the SVM by the tree-based approach included some, but not all, of the variables that showed differences between amyloid positive and amyloid negative subjects in the univariate analyses. Similarly, some of the features selected did not show statistically significant univariate group differences, although for many a trend towards lower values in amyloid positive subjects compared to amyloid negative subjects was observed. By combining the selected features derived from demographic information, neuropsychological examination, MRI measures and *APOE* ε4 genotype, we were able to classify MCI and CN subjects as amyloid positive or negative with a moderate level of accuracy.

The AUC for prediction of amyloid pathology was slightly higher in the MCI group compared to the CN group, and in line with a previous study in MCI [40]. In that study, a SVM classifier to predict amyloid pathology in subjects with MCI was also developed. Using cognitive data, hippocampal volume, *APOE* ε4 genotype and peripheral blood protein markers from the Alzheimer’s Disease Neuroimaging Initiative (ADNI) dataset, they obtained AUC = 0.80 for predicting amyloid pathology in subjects with MCI. In contrast to a previous study [12], we did not find that combining MRI markers with *APOE* ε4 genotype improved prediction of amyloid pathology in MCI over only including *APOE* ε4.

Our results in CN are comparable to the result from a similar study using data from the ADNI and a monocentric cohort [41]. In that study, a machine-learning-based classifier including demographic variables, *APOE* ε4 genotype, cognitive testing and structural MRI data reached an AUC of around 0.6 in CN subjects to predict amyloid positivity. Other studies have used combinations of demographic information, *APOE* ε4 genotype and cognitive testing (without imaging measures) to predict amyloid positivity in CN [42, 43]. They obtained positive predictive values of 0.65 and 0.63 for amyloid positivity, which was a 43–59% increase compared to the baseline prevalence in the cohort (0.41 and 0.44 respectively). In comparison, in our study we obtained a positive predictive value of 0.41 for amyloid pathology in CN, with a baseline prevalence of 0.25 in our cohort, which is a 64% increase in predictive value. To recruit 1000 CN subjects with amyloid pathology, using the classifier could reduce the number of subjects needing to undergo amyloid assessment from 3925 to 2439, which is a 38% decrease. Assuming a cost of €850 for the pre-screening (including MRI, *APOE* genotyping and cognitive testing) and €3500 for an amyloid PET scan, using the classifier for pre-screening could reduce the total screening costs by nearly €2 million in this CN population. This example is based on an optimized sum of sensitivity and specificity (Youden index). For clinical trial design, it might be more interesting to optimize the sensitivity of the classifier, which would minimize the proportion of falsely excluded amyloid positive subjects, at the cost of the positive predictive value. As can be seen in Table 4, with increasing sensitivity (and higher negative predictive value), the positive predictive value of the classifier becomes lower, which would lead to increasing costs of pre-screening.

We chose SVM as a classification method for several reasons. First, it is based on a robust strategy (i.e. maximum-margin hyper-plane), which is considered to be one of the best to reduce the prediction error in a classification task [44, 45]. Second, only few parameters need to be tuned in order to make it fully operational, making SVM relatively easy to set up and use. Finally, it is particularly well suited for the separation of two classes (in this case, amyloid positive and amyloid negative).

A strength of our study is that, unlike previous studies [40–42], we performed our study in a heterogeneous cohort, in which data acquisition protocols were not standardized and different MR scanners and acquisition parameters were used. In this heterogeneous cohort, we showed a similar predictive accuracy compared to prospective research cohorts, which used standardized data acquisition protocols. This highlights the robustness of our approach and suggests that the results may also be generalizable to other cohorts. This will need to be tested in future studies. Our results may be of interest for studies recruiting subjects from parent cohorts to be included in (secondary) prevention studies targeting anti-amyloid therapeutics [4]. Our findings suggest that for individuals with MCI, screening for amyloid positivity can best be done by age and *APOE* ε4 genotype, with limited added value of MRI. In CN, MRI measures have an added value above the other markers.

This study has some limitations. First, we used data acquired at various centres, which had different inclusion criteria for subjects and used different protocols for data collection. However, as already discussed, this also increased generalizability. Second, not everyone had the same measure of amyloid pathology. When possible, we used centralized analysis of the CSF Aβ_42/40_ ratio to identify amyloid positivity, which has been shown to correlate highly with PET measures of amyloid pathology [46, 47]. For data from one cohort, we only had amyloid PET data available. Although CSF and PET measures are usually in good agreement, some studies have suggested that CSF values might become abnormal earlier than PET [48, 49]. Finally, the same dataset was used to train and test the SVM classifier. Although nested *k*-fold cross-validation grants good generalizability of the SVM model [36], studies in independent datasets are needed to further validate our results.

## Conclusions

Amyloid pathology is associated with structural MRI changes in AD typical regions in CN subjects and in subjects with MCI. We developed a classifier that can predict amyloid pathology at a single subject level using a combination of easily obtainable, non-invasive measures. Our results are of interest for trial designers who intend to recruit a large number of amyloid positive subjects. Implementing pre-screening procedures consisting of simple, non-invasive tests could substantially reduce screening failure rates. In future studies, the classifier might be improved by adding data from other minimally invasive tests, such as blood proteins and genetic markers [40]. In the EMIF-AD MBD study, plasma proteomics and metabolomics, and genomics and epigenomics, will also be analysed.

## Additional files

Additional file 1: Additional Tables S1–S8 (PDF 264 kb)

Additional file 2: Additional Figures S1–S3 (PDF 770 kb)

## Abbreviations

AD: Alzheimer’s disease
*APOE*: Apolipoprotein E
CN: Cognitively normal
CSF: Cerebrospinal fluid
EMIF-AD MBD: European Medical Information Framework for Alzheimer’s Disease Multimodal Biomarker Discovery
GCA: Global cortical atrophy visual rating
MCI: Mild cognitive impairment
MMSE: Mini-Mental State Examination
MRI: Magnetic resonance imaging
MTA: Medial temporal lobe atrophy visual rating
PET: Positron emission tomography
SVM: Support vector machine
TIV: Total intracranial volume
