# Supplementary material for: MRI predictors of amyloid pathology: results from the EMIF-AD Multimodal Biomarker Discovery study
Source: Alzheimers Res Ther. 2018 Sep 27;10:100. doi: 10.1186/s13195-018-0428-1 (PMC6161396; doi:10.1186/s13195-018-0428-1)
Supplement: Supplementary file 1 — Additional Tables S1–S8 (PDF 264 kb) [file 13195_2018_428_MOESM1_ESM.pdf]

MRI predictors of amyloid pathology: results from the EMIF-AD multimodal biomarker discovery study

M. ten Kate, A. Redolfi, E. Peira, I. Bos, S.J. Vos, R. Vandenberghe, S. Gabel, J. Schaeverbeke, P. Scheltens, O. Blin, J.C. Richardson, R. Bordet, A. Wallin, C. Eckerstrom, J.L. Molinuevo, S. Engelborghs, C. Van Broeckhoven, P. Martinez-Lage, J. Popp, M. Tsolaki, F. Verhey, A.L. Baird, C. Legido-Quigley, L. Bertram, V. Dobricic, H. Zetterberg, S. Lovestone, J. Streffer, S. Bianchetti, G.P. Novak, J. Revillard, M.F. Gordon, Z. Xie, V. Wottschel, G. Frisoni, P.J. Visser, F. Barkhof

Supplementary Tables

Table S1: Priority neuropsychological test per domain per cohort

Cohort	Memory	Attention	Executive functioning	Language	Visuoconstruction
Amsterdam	RAVLT	TMT-A	TMT-B	Animal fluency	Copy of Rey complex figure
Antwerp	RBANs, memory<br>WMS	RBANs, concentration<br>WMS, working memory	TMT-B	RBANs, language<br>BNT-60	RBANs, visuoconstruction<br>Copy of Rey complex figure
DESCRIPA	RAVLT	TMT-A	TMT-B	Animal fluency	Copy of Rey complex figure
EDAR	CERAD word list	TMT-A	TMT-B	Animal fluency	Copy CERAD figures
GAP	FCSR	TMT-A	TMT-B	BNT-60	Copy of Rey complex figure
Gothenburg	RAVLT	TMT-A	TMT-B	Letter fluency	Copy of Rey complex figure
IDIBAPS	FCSR	TMT-A	TMT-B	Animal fluency	Copy of Rey complex figure
Lausanne	RI-48 cued recall test	TMT-A	TMT-B	Animal fluency	Copy CERAD figures
Leuven	RAVLT	TMT-A	TMT-B	Animal fluency	-
Pharmacog	RAVLT	TMT-A	TMT-B	Category fluency, sum of three	-
Sant Pau	-	-	-	-	-

BNT: Boston Naming Test; CERAD: Consortium to Establish a Registry for AD; FCSR: Free and Cued Selective Reminding Test; RAVLT: Rey Auditory Verbal Learning Test; RBANs = Repeatable Battery for the Assessment of Neuropsychological status; TMT-A: Trail Making Test, part A; TMT-B: Trail Making Test, part B; WMS: Wechsler Memory Scale.

Table S2: Number of amyloid positive and negative subjects per diagnosis per cohort

Diagnosis	Cognitively normal		Mild Cognitive Impairment		Alzheimer type dementia		Total	
Amyloid status	Negative	Positive	Negative	Positive	Negative	Positive	Negative	Positive
Amsterdam	15 (50)	15 (50)	41 (50)	41 (50)	1 (2)	59 (98)	57 (33)	115 (67)
Antwerp	-	-	9 (18)	41 (82)	-	7 (100)	9 (16)	48 (84)
DESCRIPA	4 (67)	2 (33)	6 (75)	2 (25)	-	-	10 (71)	4 (29)
EDAR	13 (93)	1 (7)	11 (44)	14 (56)	3 (16)	16 (84)	27 (47)	31 (53)
GAP	26 (68)	12 (32)	-	-	-	-	26 (68)	12 (32)
Gothenburg	27 (56)	21 (44)	26 (57)	20 (43)	-	-	53 (56)	41 (44)
IDIBAPS	27 (68)	13 (32)	-	14 (100)	-	14 (100)	27 (40)	41 (60)
Lausanne	7 (70)	3 (30)	11 (42)	15 (58)	-	-	18 (50)	18 (50)
Leuven	153 (85)	26 (15)	-	-	-	-	153 (85)	26 (15)
Pharmacog	-	-	56 (38)	91 (62)	-	-	56 (38)	91 (62)
Sant Pau	-	-	-	-	-	-	-	-
Total	272 (75)	93 (25)	160 (40)	238 (60)	4 (4)	96 (96)	436 (51)	427 (49)

Data are displayed as count (percentage). Numbers are based on subjects with a good quality T1-weighted MRI scan.

Table S3: Available MRI sequences for each cohort and acquisition parameters for 3DT1.

	Contributed sequences			Scanner, field strength	Acquisition parameters 3D T1					
Cohort	3DT1	FLAIR	T2* / SWI		Plane	TR (ms)	TI (ms)	TE (ms)	FA	Voxel size (mm)
Amsterdam	Yes	Yes	Yes	GE 3T	3D sagittal	7.8	3.0	450	12°	0.98 x 0.98 x 1.00
Antwerp	Yes	Yes	Yes	Siemens 3T	3D transversal	1910	1100	3.37	15°	1.00 x 1.00 x 1.00
DESCRIPA site 1	Only 2D	Yes	-	Philips 1.5T	-	-	-	-	-	-
DESCRIPA site 2	Yes	Yes	Yes	Siemens 1T	3D coronal	15	300	7	15°	0.98 x 0.98 x 1.49
EDAR site 1.1	Yes	Yes	Yes	Siemens 1.5T	3D sagittal	2700	950	5.17	8°	1.00 x 1.00 x 1.50
EDAR site 1.2	Yes	Yes	Yes	GE 3T	3D sagittal	7.8	3.0	450	12°	0.94 x 0.94 x 1.00
EDAR site 2	Yes	Yes	-	Philips 1.5T	3D sagittal	20	-	3	30°	1.00 x 1.00 x 2.00
EDAR site 3	Yes	Yes	Yes	Philips 3T	3D sagittal	8.2	-	3.8	8°	1.00 x 1.00 x 1.00
GAP	Yes	Yes	-	Siemens 3T	3D sagittal	2300	900	2.86	9°	1.25 x 1.25 x 1.25
Gothenburg	Yes	Yes	-	Siemens 1.5T	3D coronal	1610	820	2.38	15°	0.49 x 0.49 x 1.00
IDIBAPS	Yes	Yes	-	Siemens 3T	3D sagittal	2300	900	2.98	9°	1.00 x 1.00 x 1.00
Lausanne	Yes	Yes	Yes	Siemens 3T	3D sagittal	2300	900	2.98	9°	1.00 x 1.00 x 1.00
Leuven	Yes	Yes	-	Philips 3T	3D coronal	9.6	-	4.6	8°	0.98 x 0.98 x 1.20
Pharmacog site 1	Yes	Yes	Yes	Siemens 3T	3D sagittal	2300	900	2.83	9°	1.00 x 1.00 x 1.00
Pharmacog site 2	Yes	Yes	Yes	Siemens 3T	3D sagittal	2300	900	2.98	9°	1.00 x 1.00 x 1.00
Pharmacog site 3	Yes	Yes	Yes	Siemens 3T	3D sagittal	2300	900	2.98	9°	1.00 x 1.00 x 1.00
Pharmacog site 4	Yes	Yes	Yes	Philips 3T	3D sagittal	2300	900	3.16	9°	1.00 x 1.00 x 1.00
Pharmacog site 5	Yes	Yes	Yes	Philips 3T	3D sagittal	2300	900	3.16	9°	1.00 x 1.00 x 1.00
Pharmacog site 6	Yes	Yes	Yes	GE 3T	3D sagittal	2300	900	2.86	9°	1.00 x 1.00 x 1.00
Pharmacog site 7	Yes	Yes	Yes	Siemens 3T	3D sagittal	2300	900	2.98	9°	1.00 x 1.00 x 1.00
Pharmacog site 8	Yes	Yes	Yes	Siemens 3T	3D sagittal	2300	900	2.03	9°	1.00 x 1.00 x 1.00
Sant Pau	-	-	-	-	-	-	-	-	-	-

FA: flip angle; TR: repetition time; TI: inversion time; TE: echo time

Table S4: Comparison between subjects who were included and excluded based on time difference between amyloid assessment and MRI

Diagnosis	Cognitively normal		Mild Cognitive Impairment		Alzheimer type dementia	
Included / Excluded	Included	Excluded	Included	Excluded	Included	Excluded
N, % within group	337 (92)	28 (8)	374 (94)	23 (6)	98 (98)	2 (2)
Age, yrs	66.5 ± 7.2	64.8 ± 6.9	69.1 ± 7.5	66.3 ± 9.1	67.0 ± 7.6	70.0 ± 19.8
Male gender	167 (50)	9 (33)	178 (48)	10 (44)	51 (52)	1 (50)
Education, yrs	13.1 ± 3.6	14.2 ± 3.4	11.0 ± 3.8	11.7 ± 3.4	11.1 ± 3.3	9.5 ± 4.9
MMSE	28.9 ± 1.1	29.3 ± 0.8	26.4 ± 2.5	27.6 ± 2.2 [A]	22.5 ± 4.0	25.5 ± 2.1
APOE ε4 genotype	142 (42)	9 (32)	187 (50)	9 (39)	68 (69)	1 (50)

Data are presented as mean ± SD or count (%). [A] $p < 0.05$, [B] $p < 0.01$, [C] $p < 0.001$ different between included and excluded subjects within diagnostic group.

Table S5: Comparison between subjects who were included and excluded based on time difference between cognitive assessment and MRI

Diagnosis	Cognitively normal		Mild cognitive impairment		Alzheimer type dementia	
Included / Excluded	Included	Excluded	Included	Excluded	Included	Excluded
N, % within group	320 (88)	44 (12)	345 (87)	52 (13)	97 (97)	3 (3)
Age, yrs	66.6 ± 7.3	65.0 ± 7.1	69.2 ± 7.4	67.0 ± 9.0	67.3 ± 7.8	60.1 ± 8.9
Male gender	160 (50)	16 (36)	166 (48)	22 (42)	48 (51)	3 (100)
Education, yrs	13.1 ± 3.6	14.2 ± 3.4 [A]	11.0 ± 3.9	11.1 ± 2.9	11.0 ± 3.4	12.8 ± 0.6 [B]
MMSE	28.8 ± 1.1	29.3 ± 0.8 [C]	26.4 ± 2.5	27.0 ± 2.7	22.5 ± 4.1	25.0 ± 2.6
APOE ε4 genotype	134 (42)	17 (39)	176 (51)	20 (38)	66 (68)	3 (100)

Data are presented as mean ± SD or count (%). [A] $p < 0.05$, [B] $p < 0.01$, [C] $p < 0.001$ different between included and excluded subjects within diagnostic group.

Table S6: Cortical thickness measures according to diagnosis and amyloid status

Diagnosis	Cognitively Normal		Mild Cognitive Impairment		Alzheimer type dementia	
Amyloid status	Negative	Positive	Negative	Positive	Negative	Positive
L superior frontal	2.49 (0.03)	2.48 (0.03)	**2.47 (0.03)**	**2.43 (0.03)** [A]	2.35 (0.10)	2.40 (0.03)
R superior frontal	2.48 (0.02)	2.48 (0.03)	2.46 (0.02)	2.44 (0.03)	2.38 (0.10)	2.40 (0.03)
L rostral middle frontal	2.19 (0.03)	2.17 (0.03)	2.16 (0.03)	2.13 (0.03)	2.16 (0.10)	2.12 (0.03)
R rostral middle frontal	2.16 (0.02)	2.17 (0.03)	2.15 (0.03)	2.12 (0.03)	2.10 (0.09)	2.12 (0.03)
L caudal middle frontal	2.33 (0.03)	2.32 (0.03)	**2.29 (0.03)**	**2.24 (0.03)** [A]	2.27 (0.11)	2.21 (0.03)
R caudal middle frontal	2.34 (0.03)	2.31 (0.03)	**2.29 (0.03)**	**2.24 (0.03)** [A]	2.20 (0.11)	2.20 (0.03)
L pars opercularis	2.38 (0.03)	2.39 (0.03)	2.35 (0.03)	2.32 (0.03)	2.24 (0.10)	2.33 (0.03)
R pars opercularis	2.39 (0.02)	2.38 (0.03)	2.36 (0.02)	2.36 (0.02)	2.24 (0.11)	2.35 (0.03)
L pars orbitalis	2.50 (0.04)	2.50 (0.04)	2.46 (0.04)	2.44 (0.04)	2.58 (0.15)	2.45 (0.04)
R pars orbitalis	2.51 (0.04)	2.49 (0.04)	2.44 (0.04)	2.46 (0.04)	2.24 (0.14)	2.41 (0.04)
L pars triangularis	2.24 (0.03)	2.22 (0.03)	**2.22 (0.03)**	**2.18 (0.03)** [A]	2.20 (0.11)	2.19 (0.03)
R pars triangularis	2.24 (0.03)	2.26 (0.03)	2.22 (0.03)	2.20 (0.03)	2.04 (0.10)	2.19 (0.03)
L lateral orbitofrontal	2.51 (0.02)	2.49 (0.03)	2.47 (0.02)	2.47 (0.02)	2.48 (0.11)	2.45 (0.03)
R lateral orbitofrontal	2.49 (0.04)	2.49 (0.04)	2.46 (0.04)	2.46 (0.04)	2.41 (0.11)	2.47 (0.04)
L medial orbitofrontal	2.33 (0.03)	2.32 (0.03)	2.32 (0.03)	2.32 (0.03)	2.22 (0.11)	2.30 (0.03)
R medial orbitofrontal	2.28 (0.03)	2.27 (0.03)	2.28 (0.03)	2.29 (0.03)	2.26 (0.13)	2.29 (0.03)
L precentral	2.27 (0.05)	2.28 (0.06)	2.26 (0.05)	2.23 (0.05)	2.19 (0.12)	2.21 (0.06)
R precentral	2.26 (0.04)	2.26 (0.05)	2.25 (0.04)	2.22 (0.05)	2.08 (0.11)	2.21 (0.05)
L paracentral	2.10 (0.06)	2.11 (0.06)	2.14 (0.06)	2.10 (0.06)	2.16 (0.13)	2.11 (0.06)
R paracentral	2.16 (0.06)	2.14 (0.06)	2.17 (0.06)	2.13 (0.06)	2.17 (0.12)	2.16 (0.06)
L frontal pole	2.62 (0.04)	2.57 (0.05)	**2.58 (0.05)**	**2.51 (0.05)** [A]	2.18 (0.19)	2.51 (0.05)
R frontal pole	2.51 (0.04)	2.53 (0.04)	2.51 (0.04)	2.48 (0.04)	2.23 (0.20)	2.48 (0.05)
L superior parietal	1.99 (0.03)	2.00 (0.03)	**1.99 (0.03)**	**1.93 (0.03)** [B]	1.95 (0.11)	1.90 (0.03)
R superior parietal	1.97 (0.03)	1.97 (0.03)	**1.97 (0.03)**	**1.91 (0.03)** [B]	1.89 (0.11)	1.87 (0.03)
L inferior parietal	2.22 (0.02)	2.19 (0.02)	**2.21 (0.02)**	**2.12 (0.02)** [C]	2.18 (0.11)	2.06 (0.03)

R inferior parietal	2.23 (0.03)	2.21 (0.03)	**2.21 (0.03)**	**2.13 (0.03)** [C]	2.19 (0.11)	2.06 (0.03)
L supramarginal	2.28 (0.02)	2.28 (0.03)	**2.29 (0.03)**	**2.22 (0.03)** [C]	2.31 (0.11)	2.16 (0.03)
R supramarginal	2.31 (0.03)	2.28 (0.03)	**2.30 (0.03)**	**2.25 (0.03)** [B]	2.25 (0.10)	2.18 (0.03)
L postcentral	1.88 (0.03)	1.87 (0.03)	**1.87 (0.03)**	**1.83 (0.03)** [A]	1.83 (0.09)	1.83 (0.03)
R postcentral	1.86 (0.03)	1.86 (0.03)	1.85 (0.03)	1.83 (0.03)	1.74 (0.09)	1.85 (0.03)
L precuneus	2.16 (0.02)	2.14 (0.03)	**2.15 (0.02)**	**2.07 (0.02)** [C]	2.07 (0.10)	2.02 (0.03)
R precuneus	2.18 (0.02)	2.15 (0.03)	**2.17 (0.02)**	**2.10 (0.02)** [C]	2.15 (0.10)	2.03 (0.03)
L inferior temporal	2.65 (0.04)	2.61 (0.04)	**2.60 (0.04)**	**2.53 (0.04)** [B]	**2.70 (0.13)**	**2.44 (0.04)** [A]
R inferior temporal	2.69 (0.04)	2.66 (0.04)	**2.64 (0.04)**	**2.58 (0.04)** [A]	**2.76 (0.13)**	**2.51 (0.04)** [A]
L middle temporal	**2.66 (0.03)**	**2.60 (0.03)** [B]	**2.64 (0.03)**	**2.55 (0.03)** [C]	2.64 (0.12)	2.47 (0.03)
R middle temporal	**2.73 (0.03)**	**2.68 (0.03)** [A]	**2.66 (0.03)**	**2.61 (0.03)** [B]	2.71 (0.12)	2.51 (0.04)
L superior temporal	2.55 (0.03)	2.52 (0.03)	**2.52 (0.03)**	**2.46 (0.03)** [B]	2.53 (0.12)	2.38 (0.03)
R superior temporal	2.58 (0.02)	2.55 (0.03)	**2.55 (0.02)**	**2.50 (0.02)** [B]	2.57 (0.12)	2.41 (0.03)
L banks superior temporal sulcus	2.27 (0.03)	2.25 (0.03)	**2.29 (0.03)**	**2.20 (0.03)** [C]	2.2 (0.13)	2.11 (0.03)
R banks superior temporal sulcus	2.39 (0.03)	2.36 (0.03)	**2.39 (0.03)**	**2.30 (0.03)** [C]	2.34 (0.13)	2.21 (0.03)
L fusiform	2.58 (0.03)	2.54 (0.03)	**2.54 (0.03)**	**2.48 (0.03)** [B]	2.59 (0.11)	2.40 (0.03)
R fusiform	2.59 (0.03)	2.55 (0.03)	**2.53 (0.03)**	**2.47 (0.03)** [B]	2.51 (0.12)	2.38 (0.03)
L transverse temporal	2.09 (0.04)	2.12 (0.04)	2.10 (0.04)	2.10 (0.04)	2.11 (0.14)	2.08 (0.04)
R transverse temporal	2.16 (0.04)	2.20 (0.04)	2.18 (0.04)	2.17 (0.04)	1.91 (0.15)	2.13 (0.04)
L entorhinal	3.28 (0.04)	3.25 (0.05)	**3.21 (0.04)**	**3.07 (0.04)** [B]	2.97 (0.28)	2.92 (0.05)
R entorhinal	3.45 (0.05)	3.49 (0.06)	3.31 (0.05)	3.24 (0.05)	3.29 (0.30)	3.02 (0.06)
L temporal pole	3.47 (0.04)	3.47 (0.05)	3.39 (0.04)	3.38 (0.04)	3.49 (0.25)	3.34 (0.05)
R temporal pole	3.65 (0.04)	3.63 (0.05)	3.48 (0.04)	3.51 (0.04)	3.54 (0.25)	3.39 (0.05)
L parahippocampal	**2.60 (0.03)**	**2.50 (0.04)** [A]	**2.61 (0.04)**	**2.47 (0.04)** [B]	**2.90 (0.24)**	**2.34 (0.05)** [A]
R parahippocampal	2.59 (0.04)	2.53 (0.04)	2.54 (0.04)	2.47 (0.04)	2.68 (0.21)	2.32 (0.05)
L lateral occipital	2.01 (0.03)	2.02 (0.03)	**2.01 (0.03)**	**1.97 (0.03)** [A]	1.94 (0.10)	1.94 (0.03)
R lateral occipital	2.03 (0.03)	2.04 (0.03)	**2.06 (0.03)**	**2.01 (0.03)** [B]	2.01 (0.10)	1.96 (0.04)
L lingual	1.89 (0.02)	1.88 (0.02)	1.85 (0.02)	1.82 (0.02)	1.82 (0.09)	1.83 (0.03)
R lingual	1.93 (0.02)	1.92 (0.02)	**1.92 (0.02)**	**1.88 (0.02)** [B]	1.77 (0.10)	1.86 (0.02)

L cuneus	1.69 (0.03)	1.68 (0.03)	1.68 (0.03)	1.66 (0.03)	1.57 (0.10)	1.69 (0.04)
R cuneus	1.72 (0.03)	1.73 (0.03)	1.73 (0.03)	1.70 (0.03)	1.69 (0.10)	1.72 (0.04)
L pericalcarine	1.48 (0.03)	1.49 (0.03)	1.49 (0.03)	1.48 (0.03)	**1.34 (0.10)**	**1.54 (0.03)** [A]
R pericalcarine	1.52 (0.03)	1.52 (0.03)	**1.55 (0.03)**	**1.52 (0.03)** [A]	1.47 (0.10)	1.55 (0.03)
L rostral anterior cingulate	2.73 (0.03)	2.73 (0.03)	2.70 (0.03)	2.74 (0.03)	2.75 (0.18)	2.73 (0.04)
R rostral anterior cingulate	2.71 (0.03)	2.72 (0.03)	**2.67 (0.03)**	**2.75 (0.03)** [B]	2.70 (0.17)	2.73 (0.04)
L caudal anterior cingulate	2.58 (0.03)	2.55 (0.04)	2.53 (0.03)	2.56 (0.03)	2.70 (0.22)	2.54 (0.04)
R caudal anterior cingulate	2.44 (0.03)	2.40 (0.03)	2.41 (0.03)	2.44 (0.03)	2.53 (0.18)	2.45 (0.04)
L posterior cingulate	2.38 (0.02)	2.35 (0.02)	2.35 (0.02)	2.33 (0.02)	2.33 (0.11)	2.26 (0.02)
R posterior cingulate	2.31 (0.02)	2.30 (0.02)	2.31 (0.02)	2.30 (0.02)	2.32 (0.11)	2.24 (0.02)
L isthmus cingulate	2.33 (0.02)	2.31 (0.03)	2.25 (0.03)	2.21 (0.03)	2.21 (0.14)	2.13 (0.03)
R isthmus cingulate	2.24 (0.02)	2.23 (0.03)	2.20 (0.02)	2.16 (0.02)	2.33 (0.14)	2.09 (0.03)
L insula	2.90 (0.02)	2.91 (0.03)	2.89 (0.02)	2.90 (0.02)	2.79 (0.13)	2.85 (0.03)
R insula	2.89 (0.02)	2.87 (0.02)	2.86 (0.02)	2.85 (0.02)	2.72 (0.12)	2.81 (0.02)

Data are presented as estimate (standard error). Estimates were derived from linear mixed models including diagnosis x amyloid and age, gender and *APOE* ε4 genotype as covariates and cohort as random effect. *APOE*: apolipoprotein E; L: left; R: right.
[A] $p < 0.05$, [B] $p < 0.01$, [C] $p < 0.001$ compared to amyloid negative within diagnostic group.

Table S7: Surface area measures according to diagnosis and amyloid status

Diagnosis	Cognitively Normal		Mild Cognitive Impairment		Alzheimer type dementia	
Amyloid status	Negative	Positive	Negative	Positive	Negative	Positive
L superior frontal	7147 (124)	7094 (135)	7124 (127)	7032 (128)	**7991 (516)**	**6914 (140)** [A]
R superior frontal	7010 (124)	6943 (135)	**6988 (127)**	**6805 (128)** [A]	7676 (523)	6752 (141)
L rostral middle frontal	5723 (129)	5661 (138)	**5701 (131)**	**5496 (132)** [A]	5805 (487)	5419 (143)
R rostral middle frontal	5909 (137)	5822 (146)	5857 (139)	5727 (140)	6501 (502)	5574 (150)
L caudal middle frontal	2304 (53)	2227 (59)	2316 (54)	2255 (55)	2356 (252)	2204 (62)
R caudal middle frontal	2132 (53)	2051 (59)	2138 (54)	2072 (55)	2267 (236)	2033 (61)
L pars opercularis	1619 (38)	1610 (43)	1650 (39)	1608 (40)	1690 (186)	1584 (45)
R pars opercularis	1380 (28)	1392 (33)	**1418 (30)**	**1363 (30)** [A]	1476 (151)	1320 (35)
L pars orbitalis	623 (11)	622 (12)	623 (11)	606 (12)	634 (53)	581 (13)
R pars orbitalis	769 (13)	766 (15)	768 (13)	752 (13)	835 (66)	721 (15)
L pars triangularis	1260 (26)	1253 (29)	1275 (27)	1240 (27)	1440 (124)	1211 (30)
R pars triangularis	1440 (28)	1464 (33)	1459 (30)	1434 (30)	1518 (152)	1400 (35)
L lateral orbitofrontal	2550 (44)	2539 (49)	**2585 (45)**	**2505 (46)** [A]	2774 (203)	2426 (51)
R lateral orbitofrontal	2500 (58)	2456 (61)	2494 (59)	2470 (59)	**2782 (200)**	**2379 (63)** [A]
L medial orbitofrontal	1840 (40)	1825 (43)	1832 (41)	1814 (41)	2104 (166)	1793 (45)
R medial orbitofrontal	1827 (29)	1798 (32)	1819 (30)	1782 (30)	**2055 (140)**	**1770 (34)** [A]
L precentral	4997 (101)	4961 (107)	5061 (102)	4985 (103)	5274 (356)	4976 (110)
R precentral	5045 (105)	5014 (110)	5039 (106)	5018 (107)	**5963 (352)**	**5058 (113)** [B]
L paracentral	1369 (28)	1390 (31)	1388 (29)	1385 (29)	1512 (133)	1348 (33)
R paracentral	**1555 (32)**	**1618 (36)** [A]	1571 (33)	1587 (33)	1653 (150)	1561 (37)
L frontal pole	208 (5)	205 (5)	201 (5)	198 (5)	182 (23)	201 (5)
R frontal pole	**284 (6)**	**270 (6)** [B]	273 (6)	269 (6)	**345 (29)**	**275 (7)** [A]
L superior parietal	5379 (108)	5294 (117)	5343 (110)	5355 (111)	5789 (423)	5253 (121)
R superior parietal	5442 (105)	5359 (113)	5396 (107)	5312 (108)	6010 (410)	5230 (117)
L inferior parietal	4683 (92)	4606 (100)	4721 (94)	4586 (95)	**5107 (395)**	**4307 (105)** [A]

R inferior parietal	5659 (106)	5571 (117)	**5565 (109)**	**5375 (110)** [A]	**6237 (463)**	**5199 (122)** [A]
L supramarginal	3966 (73)	3891 (82)	3909 (76)	3867 (77)	3751 (345)	3792 (86)
R supramarginal	3676 (65)	3690 (74)	3748 (68)	3671 (68)	3245 (327)	3537 (78)
L postcentral	4313 (73)	4252 (80)	4280 (74)	4315 (75)	4455 (315)	4308 (83)
R postcentral	4132 (68)	4112 (75)	4147 (70)	4172 (71)	4162 (304)	4096 (79)
L precuneus	3786 (63)	3737 (70)	3790 (65)	3758 (65)	3907 (285)	3610 (73)
R precuneus	3948 (56)	3913 (65)	3973 (59)	3928 (60)	3869 (307)	3728 (69)
L inferior temporal	**3332 (81)**	**3191 (87)** [A]	3269 (82)	3196 (83)	**4070 (316)**	**3007 (90)** [B]
R inferior temporal	**3138 (66)**	**2971 (72)** [B]	3123 (67)	3059 (68)	**3499 (296)**	**2841 (76)** [A]
L middle temporal	**3073 (57)**	**2954 (63)** [A]	**3022 (58)**	**2931 (59)** [A]	3222 (257)	2823 (65)
R middle temporal	**3399 (58)**	**3244 (64)** [B]	**3356 (59)**	**3218 (60)** [B]	3523 (261)	3085 (67)
L superior temporal	3773 (64)	3741 (70)	**3779 (65)**	**3645 (66)** [B]	3778 (271)	3618 (73)
R superior temporal	3575 (63)	3535 (68)	**3589 (64)**	**3487 (65)** [A]	3837 (251)	3394 (70)
L banks superior temporal sulcus	1048 (21)	1047 (25)	**1049 (22)**	**999 (23)** [A]	986 (113)	987 (26)
R banks superior temporal sulcus	974 (17)	949 (20)	**965 (18)**	**923 (18)** [B]	929 (92)	867 (21)
L fusiform	3243 (62)	3162 (68)	3172 (64)	3122 (64)	2879 (265)	3092 (71)
R fusiform	3159 (49)	3099 (56)	**3101 (51)**	**3006 (52)** [A]	2945 (252)	2909 (59)
L transverse temporal	**469 (11)**	**451 (12)** [A]	**464 (11)**	**443 (11)** [A]	447 (49)	440 (13)
R transverse temporal	338 (8)	347 (9)	351 (8)	342 (8)	**417 (36)**	**329 (9)** [A]
L entorhinal	411 (14)	399 (15)	413 (14)	399 (14)	329 (51)	373 (15)
R entorhinal	**369 (11)**	**341 (12)** [B]	**361 (11)**	**340 (12)** [A]	381 (52)	347 (13)
L temporal pole	472 (9)	465 (10)	464 (9)	461 (9)	447 (42)	437 (10)
R temporal pole	416 (7)	407 (9)	418 (8)	414 (8)	405 (42)	411 (9)
L parahippocampal	701 (10)	708 (13)	676 (11)	674 (11)	551 (67)	666 (14)
R parahippocampal	683 (9)	681 (12)	664 (10)	651 (10)	**791 (63)**	**648 (13)** [A]
L lateral occipital	4754 (83)	4735 (92)	**4874 (86)**	**4660 (87)** [B]	5142 (371)	4643 (96)
R lateral occipital	4505 (85)	4466 (93)	4647 (87)	4535 (88)	**5603 (356)**	**4471 (96)** [B]
L lingual	3050 (45)	2970 (53)	3009 (48)	2938 (48)	2735 (251)	2877 (56)
R lingual	3050 (37)	2993 (47)	**3059 (41)**	**2949 (42)** [A]	3365 (247)	2941 (50)

L cuneus	1419 (24)	1409 (28)	1445 (25)	1418 (26)	1562 (129)	1369 (30)
R cuneus	1495 (26)	1465 (29)	1495 (27)	1484 (27)	1666 (133)	1431 (31)
L pericalcarine	1330 (29)	1322 (33)	1353 (30)	1339 (30)	1533 (154)	1318 (35)
R pericalcarine	1504 (27)	1494 (32)	1519 (29)	1486 (29)	1731 (163)	1477 (35)
L rostral anterior cingulate	864 (20)	837 (23)	874 (20)	860 (21)	979 (105)	814 (24)
R rostral anterior cingulate	700 (17)	678 (20)	697 (18)	693 (18)	672 (95)	669 (21)
L caudal anterior cingulate	667 (15)	643 (18)	671 (16)	655 (16)	543 (92)	638 (19)
R caudal anterior cingulate	776 (15)	757 (19)	785 (16)	781 (16)	837 (99)	756 (20)
L posterior cingulate	1161 (20)	1129 (23)	1168 (21)	1129 (21)	1063 (113)	1125 (25)
R posterior cingulate	1196 (18)	1195 (22)	**1216 (20)**	**1169 (20)** [A]	1185 (114)	1142 (24)
L isthmus cingulate	1053 (18)	1055 (22)	1062 (20)	1040 (20)	1302 (113)	1083 (24)
R isthmus cingulate	981 (19)	984 (22)	1002 (20)	977 (20)	1088 (102)	975 (23)
L insula	2203 (37)	2198 (40)	2206 (38)	2193 (38)	**2487 (148)**	**2163 (42)** [B]
R insula	2264 (47)	2256 (50)	2283 (47)	2260 (48)	**2719 (170)**	**2227 (51)** [A]

Data are presented as estimate (standard error). Estimates were derived from linear mixed models including diagnosis x amyloid and age, gender and *APOE*-ε4 as covariates and cohort as random effect. *APOE*: apolipoprotein E; L: left; R: right.
[A] $p < 0.05$, [B] $p < 0.01$, [C] $p < 0.001$ compared to amyloid negative within diagnostic group.

Table S8: Features considered and selected for classifier

	Cognitively normal	Mild Cognitive Impairment	Whole sample
Feature	Feature importance		
**Demographic**			
- Age	17 (0.009)	18 (0.020)	27 (0.015)
- Sex			
- Education			
**Neuropsychology**			
- MMSE		7 (0.033)	10 (0.034)
- CDR Total			
- Memory immediate	4 (0.023)		31 (0.015)
- Memory delayed		37 (0.009)	
- Language	9 (0.015)		
- Attention			
- Executive functioning	33 (0.007)		
- Visuoconstruction	37 (0.006)		
**MRI Visual rating**			
- MTA			2 (0.051)
- MTA abnormal			8 (0.035)
- GCA		26 (0.009)	13 (0.031)
- Parietal			12 (0.032)
- Fazekas	44 (0.005)		
- Lacunes	27 (0.007)		
**MRI cortical thickness**			
- L superior frontal			
- R superior frontal		20 (0.015)	19 (0.016)
- L rostral middle frontal		11 (0.026)	
- R rostral middle frontal		9 (0.029)	
- L caudal middle frontal		38 (0.009)	
- R caudal middle frontal			

	Cognitively normal	Mild Cognitive Impairment	Whole sample
Feature	Feature importance		
**Genetic**			
- *APOE* ε4 carrier	1 (0.071)	1 (0.121)	1 (0.135)
**MRI subcortical volumes**			
- L hippocampus	12 (0.011)	6 (0.036)	11 (0.034)
- R hippocampus	8 (0.018)	2 (0.059)	3 (0.051)
- L amygdala	5 (0.023)		15 (0.028)
- R amygdala	7 (0.019)	15 (0.020)	9 (0.035)
- L thalamus		33 (0.009)	
- R thalamus			
- L caudate			
- R caudate	28 (0.007)	29 (0.009)	30 (0.015)
- L putamen	10 (0.015)		24 (0.015)
- R putamen	11 (0.012)		
- L pallidum	14 (0.010)		
- R pallidum			
- L accumbens	2 (0.036)		25 (0.015)
- R accumbens	3 (0.031)		23 (0.016)
**MRI other**			
- AD signature ROI	25 (0.007)	10 (0.028)	32 (0.008)
**MRI surface area**			
- L superior frontal			
- R superior frontal			
- L rostral middle frontal			
- R rostral middle frontal			
- L caudal middle frontal			
- R caudal middle frontal			

- L pars opercularis		13 (0.021)			- L pars opercularis	39 (0.006)		
- R pars opercularis		28 (0.012)			- R pars opercularis			
- L pars orbitalis					- L pars orbitalis			
- R pars orbitalis					- R pars orbitalis			
- L pars triangularis		24 (0.015)			- L pars triangularis			
- R pars triangularis	23 (0.008)				- R pars triangularis			
- L lateral orbitofrontal					- L lateral orbitofrontal			
- R lateral orbitofrontal	46 (0.005)				- R lateral orbitofrontal			
- L medial orbitofrontal		36 (0.009)			- L medial orbitofrontal			
- R medial orbitofrontal		19 (0.015)			- R medial orbitofrontal	30 (0.007)		
- L precentral					- L precentral	20 (0.009)		
- R precentral					- R precentral			
- L paracentral		17 (0.020)			- L paracentral	13 (0.011)		28 (0.015)
- R paracentral					- R paracentral	6 (0.020)		
- L frontal pole					- L frontal pole		32 (0.009)	
- R frontal pole					- R frontal pole	41 (0.006)		
- L superior parietal					- L superior parietal			
- R superior parietal					- R superior parietal	24 (0.007)		
- L inferior parietal		14 (0.020)			- L inferior parietal			
- R inferior parietal			17 (0.016)		- R inferior parietal			35 (0.007)
- L supramarginal					- L supramarginal			
- R supramarginal		31 (0.009)			- R supramarginal			
- L postcentral					- L postcentral			
- R postcentral					- R postcentral			
- L precuneus		23 (0.015)	33 (0.008)		- L precuneus			
- R precuneus					- R precuneus			
- L inferior temporal					- L inferior temporal			
- R inferior temporal	45 (0.005)				- R inferior temporal	26 (0.007)		
- L middle temporal	16 (0.009)		22 (0.016)		- L middle temporal			
- R middle temporal		16 (0.020)			- R middle temporal	36 (0.007)		6 (0.044)
- L superior temporal	32 (0.007)	12 (0.024)	21 (0.016)		- L superior temporal			
- R superior temporal	35 (0.007)	30 (0.009)	16 (0.016)		- R superior temporal			
- L banks superior temporal		3 (0.052)	14 (0.028)		- L banks superior temporal			
- R banks superior temporal					- R banks superior temporal	47 (0.005)		

- L fusiform			20 (0.016)		- L fusiform			
- R fusiform		25 (0.015)			- R fusiform			
- L transverse temporal					- L transverse temporal	42 (0.006)		
- R transverse temporal					- R transverse temporal			
- L entorhinal		5 (0.043)	26 (0.015)		- L entorhinal			
- R entorhinal			4 (0.045)		- R entorhinal	18 (0.009)		
- L temporal pole					- L temporal pole			
- R temporal pole					- R temporal pole			
- L parahippocampal	15 (0.002)	8 (0.032)	7 (0.044)		- L parahippocampal			
- R parahippocampal	21 (0.008)	21 (0.015)			- R parahippocampal			
- L lateral occipital					- L lateral occipital			
- R lateral occipital					- R lateral occipital			
- L lingual		34 (0.009)			- L lingual			
- R lingual					- R lingual			
- L cuneus					- L cuneus			
- R cuneus					- R cuneus			
- L pericalcarine	29 (0.007)				- L pericalcarine			
- R pericalcarine	31 (0.007)	27 (0.012)			- R pericalcarine			
- L rostral anterior cingulate					- L rostral anterior cingulate			
- R rostral anterior cingulate					- R rostral anterior cingulate			
- L caudal anterior cingulate					- L caudal anterior cingulate	43 (0.005)		
- R caudal anterior cingulate	34 (0.007)				- R caudal anterior cingulate			
- L posterior cingulate	38 (0.006)				- L posterior cingulate	40 (0.006)	35 (0.009)	
- R posterior cingulate					- R posterior cingulate			
- L isthmus cingulate		4 (0.045)	5 (0.044)		- L isthmus cingulate			
- R isthmus cingulate					- R isthmus cingulate			
- L insula		22 (0.015)	34 (0.007)		- L insula	19 (0.009)		
- R insula			18 (0.016)		- R insula	22 (0.008)		

In the table are presented the features considered for the machine-learning, organized by type of measurement. The numbers represent for each sample (CN, MCI, whole sample) the features selected ranked by most important. Averaged feature weights are reported in brackets. Those features without a number were not selected.
