# Supplementary material for: MRI predictors of amyloid pathology: results from the EMIF-AD Multimodal Biomarker Discovery study
Source: Alzheimers Res Ther. 2018 Sep 27;10:100. doi: 10.1186/s13195-018-0428-1 (PMC6161396; doi:10.1186/s13195-018-0428-1)
Supplement: Supplementary file 2 — Additional Figures S1–S3 (PDF 770 kb) [file 13195_2018_428_MOESM2_ESM.pdf]

MRI predictors of amyloid pathology: results from the EMIF-AD multimodal biomarker discovery study

M. ten Kate, A. Redolfi, E. Peira, I. Bos, S.J. Vos, R. Vandenberghe, S. Gabel, J. Schaeverbeke, P. Scheltens, O. Blin, J.C. Richardson, R. Bordet, A. Wallin, C. Eckerstrom, J.L. Molinuevo, S. Engelborghs, C. Van Broeckhoven, P. Martinez-Lage, J. Popp, M. Tsolaki, F. Verhey, A.L. Baird, C. Legido-Quigley, L. Bertram, V. Dobricic, H. Zetterberg, S. Lovestone, J. Streffer, S. Bianchetti, G.P. Novak, J. Revillard, M.F. Gordon, Z. Xie, V. Wottschel, G. Frisoni, P.J. Visser, F. Barkhof

Supplementary Figures

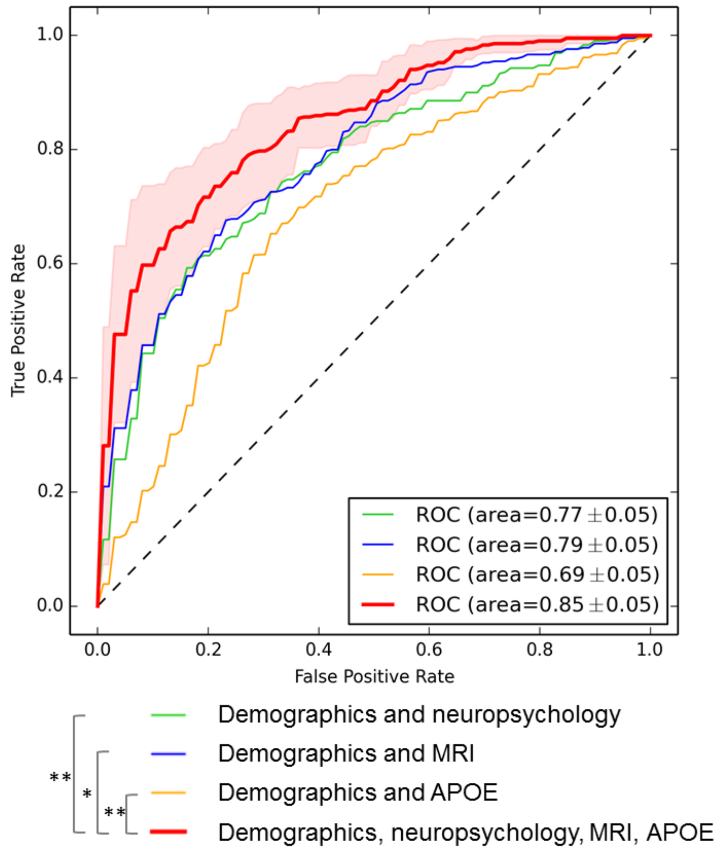

Figure S1: Classifier results in whole sample. Displayed are receiver operating characteristic (ROC) curves of support vector machine classifier to predict amyloid pathology. Red: results from the combined classifier, including demographic information, neuropsychological tests, MRI measures, and *APOE* ε4 genotype. The specific features selected are provided in Additional file 1: Table S8. In blue, yellow and green are displayed the classifier results from demographic information combined with only neuropsychology (green), or MRI measures (blue), or *APOE* ε4 genotype (yellow). ROC area differences are assessed with DeLong's test (* $p < 0.05$; ** $p < 0.001$).

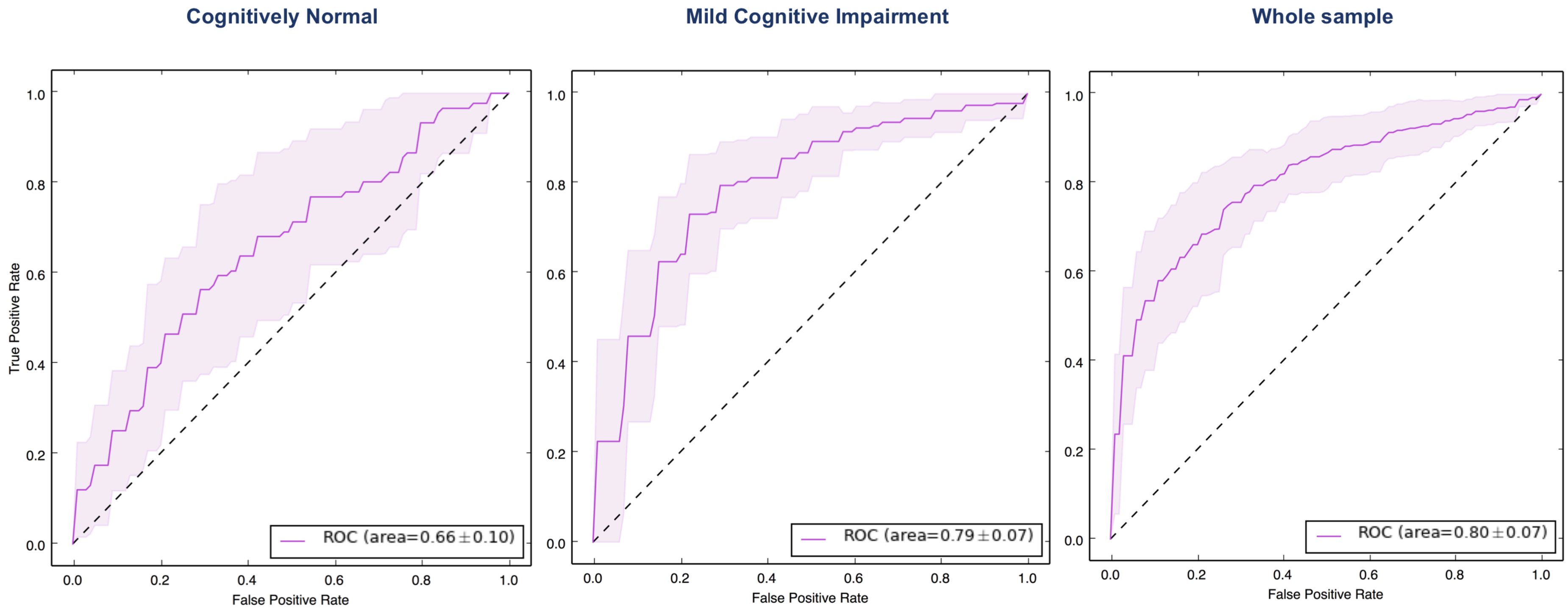

Figure S2: Classifier results including demographic, neuropsychological, APOE measures. Displayed are receiver operating characteristic (ROC) curves of support vector machine classifier to predict amyloid pathology using demographic, neuropsychological, *APOE* information in cognitively normal (left), mild cognitive impairment (middle) and whole sample (right). ROC areas of both cognitively normal and whole sample were statistically different (DeLong's test $p < 0.05$) from the ROC areas derived with the classifier including demographic, neuropsychological, MRI and *APOE* variables (displayed in Figure 1).

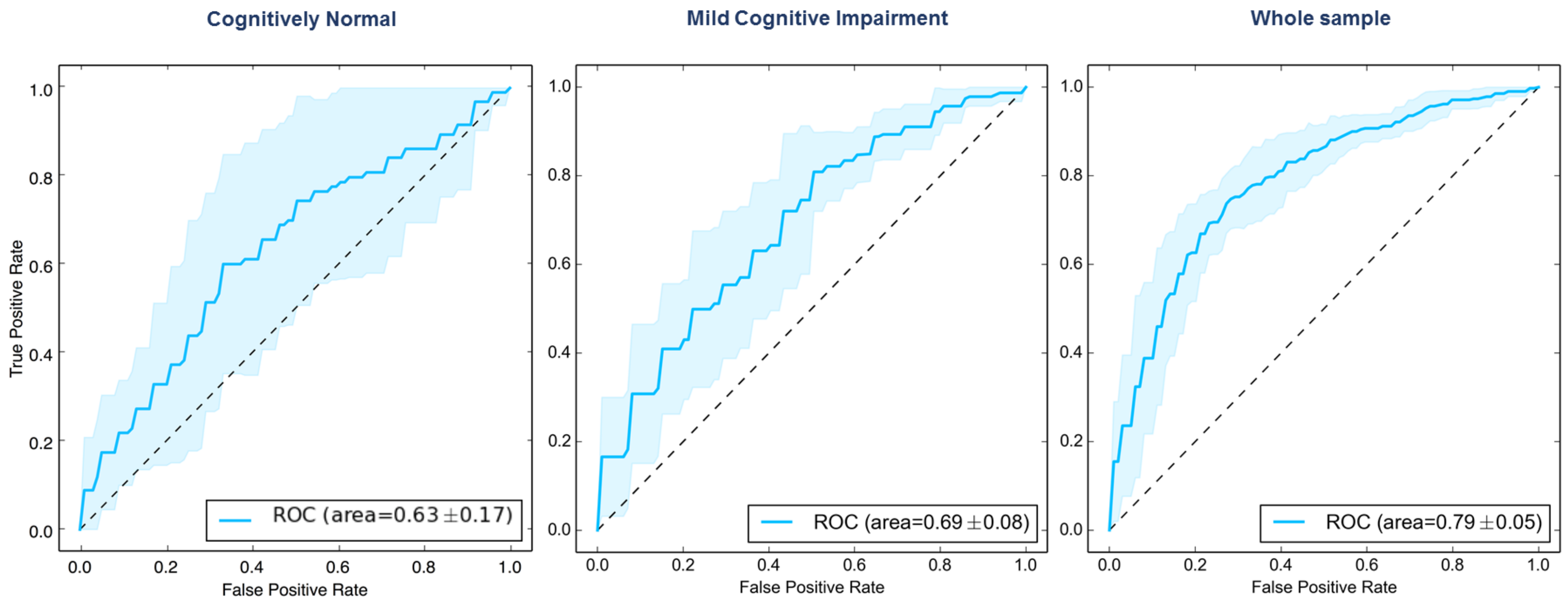

Figure S3: Classifier results only including MRI measures. Displayed are receiver operating characteristic (ROC) curves of support vector machine classifier to predict amyloid pathology using only information derived from MRI in cognitively normal (left), mild cognitive impairment (middle) and whole sample (right). ROC areas of all groups were statistically different (DeLong's test $p < 0.05$) from the ROC areas obtained with the classifier including demographic, neuropsychological, MRI and *APOE* variables (displayed in Figure 1 and Figure S1).
